# Whole-exome sequencing identifies R1279X of *MYH6* gene to be associated with congenital heart disease

**DOI:** 10.1186/s12872-018-0867-4

**Published:** 2018-07-03

**Authors:** Ehsan Razmara, Masoud Garshasbi

**Affiliations:** 1 Department of Medical Genetics, Faculty of Medical Sciences, Tarbiat Modares University, Tehran, Iran; 2 Department of Medical Genetics, DeNA laboratory, Tehran, Iran

**Keywords:** *MYH6*, Congenital heart disease, ASD type III, Nonsense mutation, WES

## Abstract

**Background:**

Myosin VI, encoded by *MYH6*, is expressed dominantly in human cardiac atria and plays consequential roles in cardiac muscle contraction and comprising the cardiac muscle thick filament. It has been reported that the mutations in the *MYH6* gene associated with sinus venosus atrial septal defect (ASD type III), hypertrophic (HCM) and dilated (DCM) cardiomyopathies.

**Methods:**

Two patients in an Iranian family have been identified who affected to Congenital Heart Disease (CHD). The male patient, besides CHD, shows that the thyroglossal sinus, refractive errors of the eye and mitral stenosis. The first symptoms emerged at the birth and diagnosis based on clinical features was made at about 5 years. The family had a history of ASD. For recognizing mutated gene (s), whole exome sequencing (WES) was performed for the male patient and variants were analyzed by autosomal dominant inheritance mode.

**Results:**

Eventually, by several filtering processes, a mutation in *MYH6* gene (NM_002471.3), c.3835C > T; R1279X, was identified as the most likely disease-susceptibility variant and then confirmed by Sanger sequencing in the family. The mutation frequency was checked out in the local databases. This mutation results in the elimination of the 660 amino acids in the C-terminal of Myosin VI protein, including the vital parts of the coiled-coil structure of the tail domain.

**Conclusions:**

Our study represents the first case of Sinus venosus defect caused directly by *MYH6* stop codon mutation. Our data indicate that by increase haploinsufficiency of myosin VI, c.3835C > T mutation with reduced penetrance could be associated with CHD.

**Electronic supplementary material:**

The online version of this article (10.1186/s12872-018-0867-4) contains supplementary material, which is available to authorized users.

## Background

Congenital Heart Defects (CHDs) are one of the major causes of death due to congenital malformations and show some of the more preponderant malformations among live births. It has been revealed that both familial and sporadic forms of CHDs result from mutations in several genes based on human cases and animal models [1, 2]. Based on targeted deletions studies in mice, it has been suggested that there are more than five hundred genes involved in heart disorders (Mouse Genome Informatics (http://www.informatics.jax.org)) [3]. CHDs treat greatly as a complex trait and to date, the number of familial cases has distinguished by the Mendelian segregation of single-gene mutations are so few [4].

Both inherited and non-inherited factors account for congenital heart disease (CHD). The incidence of CHD approximately is 0.4–0.6% live births and real prevalence is about 4% [5, 6]. Our knowledge about CHD’s causes and mechanisms remains restricted in spite of the advances in diagnosis and interventions. With development of whole exome/genome sequencing more CHD causing genes possibly will be clarified which will increase our insight into the genetic causes of CHD.

Numerous epidemiological studies have suggested a genetic component of CHD etiology. Approximately, 25% of CHD cases occur as a complex trait with related defects in other organs as a sporadic malformative association, Mendelian syndrome or chromosomal abnormality [7]. The rest of cases occur as isolated defects and both sporadic and familial cases that showing Mendelian patterns of inheritance, have been reported [8, 9]. To date, several diseases associated with *MYH6* mutations such as hypertrophic (HCM), dilated cardiomyopathy (DCM) and atrial septal defect (ASD) have been reported [10]. ASD is categorized as the second most common CHD and accounts for 10% of all cardiac malformations [11]. Around 80% of persistent small ASDs close spontaneously during infancy or childhood, but the large one could cause serious defects such as congestive heart failure, pulmonary vascular disease and etc. [12]. There are various types of ASD; ASD type III — sinus venosus atrial septal defect— is caused by mutations in *MYH6* [13]. It has not been identified any correlation between the stop codon mutations of *MYH6* and ASD type III but in the present study, we could detect this correlation.

In the present study, we checked out a clinically characterized family with a history of congenital heart disease. In this family, we observed an obvious autosomal-dominant inheritance with reduced penetrance (*K = 50%*). We identified a novel nonsense mutation in *MYH6*, NM_002471.3 c.3835C > T; R1279X, by WES of the patient in SH1190831 family and then this mutation was confirmed by Sanger sequencing.

## Methods

### Patients and clinical evaluations

The study protocol was approved by the local medical ethics committee of Tarbiat Modares University, Tehran, Iran. Written informed consents were obtained from all individuals. All of the patient’s clinical information and the medical histories were collected at the Department of Medical Genetics, *DeNA* Laboratory, Tehran, Iran.

We enrolled 5 members of this family in our study (two affected, two unaffected and one carrier) (Table 1). Subjects were adjusted by meticulous medical records including a complete physical examination, a 12-lead Echocardiogram (ECG), Ultrasonic cardiogram (UCG) and other relevant features such as PR, QRS interval, QT, QTc duration and QRS axis were measured. QRS axis was presumed as normal when its value was measured between − 30^°^ and + 90^°^ and was classified abnormal when out of this range. The normal range of ECG was performed based on the individual ages. For adults, a PR interval above 210 ms and an increased above 100 ms of QRS interval were thought-out prolonged [14].

**Table 1:** Clinical and Electrocardiographic Features in Members of SH1190831 Family

Member	status	Age range(year)	Symptoms	Clinical ECG diagnosis	QRS axis	Heart Rate Beats/min	Electrocardiography			Mutation
							RA (mm)	RV (mm)	LVEF (%)	
III.1^a,b^	P	7-11	ASD type III, Thyroglossal Sinus, Mitral Stenosis, Refractive Errors Of The Eye	RBBB	− 58°	75	29	34	63	WT/p.R1279X
II.2	P	57–61	sinus venosus atrial septal defect (ASD type III)	RBBB	−65°	88	35	33	59	WT/p.R1279X
II.3	H	36–40	Asymptomatic	ND	+ 42°	93	26	32	67	WT/WT
II.4	H	43–47	Asymptomatic	ND	+ 43°	85	31	30	64	WT/p.R1279X
II.5	H	40–44	Asymptomatic	ND	+ 42°	83	23	28	66	WT/WT

*NA* Not Detected, *ASD* Atrial Septal Defect, *RBBB* Right Bundle Branch Block, *P* Patient, *H* Healthy

^a^Whole exome sequence is applied to this individual

^b^Index case

### DNA extraction

Genomic DNA was isolated from peripheral blood of the family members by the *ROCHE* DNA Extraction Kit (Cat. No. 11814770001). DNA concentration was measured by Thermo Scientific™ Nanodrop 2000 (Thermo Fisher Scientific, Wilmington, DE, USA).

### NGS study

Exome capturing and high throughput sequencing (HTS) was performed on the proband (III:1). The Nextera Rapid Capture Exome kit with 340,000 probes designed against the human genome was utilized to enrich the approximately 37 Mb (214,405 exons) of the Consensus Coding Sequences (CCS) from fragmented genomic DNA. Due to limitations of the method, not all exons were fully covered and all of the pathogenic variants cannot be totally excluded. An overall coverage of 98.19% was achieved, with 2188 missing base pairs (a coding region including ±2 bp). At the next step, an end to end in-house bioinformatics pipelines including base calling, primary filtering of low-quality reads and probable artifacts, and annotation of variants were applied.

The reads were aligned to the NCBI human reference genome (gh19/NCBI37.1) with SNP & Variation Suite version 8.0 (SVS v8.0) and DNASTAR Lasergene12 (DNASTAR Inc., Madison, Wisconsin USA). Small indel detection was used with the Unified Genotyper tool from GATK tools in Galaxy online database (http://www.usegalaxy.org). The missense, nonsense, silent, and indel mutations rates were estimated by Galaxy online tool and finally were confirmed by Ivariantguide® (https://www.advaitabio.com).

Because of the Autosomal dominant nature of the mutation, for the first step, we assumed that the variant (s) should be transferred in heterozygote manner, so we excluded the homozygote variants and then several filtering steps were applied to prioritize all variants: 1) Variants in dbSNP132 (https://www.ncbi.nlm.nih.gov/projects/SNP) and 1000 Genomes Project (http://www.1000genomes.org) with allele frequencies more than 1% were excluded. 2) The rest of variants underwent further exclusion in Exome Sequencing Project (ESP) (http://evs.gs.washington.edu/EVS) and Exome Aggregation Consortium (ExAC) database. 3) The intragenic, intronic, UTRs regions and synonymous variants were excluded from later analysis. 4) The SIFT (http://sift.jcvi.org/), Provean (http://provean.jcvi.org) and Mutation Taster (http://www.mutationtaster.org) were used to predict variants pathogenicity (Table 2).

**Table 2:** Several online databases that used to confirm the pathogenicity of the R1279X mutation in *MYH6* gene

Mutation Taster	EXAC	SIFT	1000 Genome	Iranome	PROVEAN
Damaging	Not reported	Damaging	Not reported	Not reported	Damaging

All suspected pathogenic variants were checked out in HGMD (http://www.hgmd.cf.ac.uk) and ClinVar (https://www.ncbi.nlm.nih.gov/clinvar). Finally, based on family pedigree, autosomal dominant inheritance pattern and clinical information were used to evaluate identified variants. Based on the clinical information, specific attention has been paid to the 42 genes known Congenital Heart Diseases. ConSurf (http://www.consurf.tau.ac.il) database was applied to provide evolutionary conservation profile for Myosin VI protein and showing the staple role of the mutation (Fig. 2c). As well as, frequency of the mutation was checked out based on the local database (http://www.iranome.ir); all information related to the In-silico prediction such as conservation, allele frequency, and damaging prediction were depicted in Table 2.

### Mutation validation and co-segregation analysis

Sanger sequencing in forward and reverse directions was performed to validate the candidate variants found in WES and then segregation analyses were performed in the family. The primers were designed by Primer3.0 (http://bioinfo.ut.ee/primer3-0.4.0) web-based server (Table 3). We checked out the lack of SNPs in the genomic region corresponding to the 3′ ends of primers by looking through the dbSNP database. The primers specificity was checked by the in-silico-PCR tool in UCSC genome browser and Primer blast of NCBI genome browser and finally, the PCR was utilized in standard conditions and then the polymerase chain reaction (PCR) products were sequenced by ABI 730XL, using the conventional capillary system, and then the Sequences were analyzed by Genome Compiler online tool (http://www.genomecompiler.com/) to identify the alternations.

**Table 3:** Sequences of the primers used to confirm the mutation by Sanger sequencing

Patient ID	Gene	Variant	Primers
SH1190831	*MYH6*	c.3835C > T (p.R1279X)	F 5′-CACACTCACCCTTCCTGTCT-3′ R 5′-CTGAAATGAGGGGCTTGTGG-3′

## Results

### Clinical features

We identified two patients — the female and the male patients— in an Iranian family. The female patient had sinus venosus atrial septal defect, although the male patient manifested other symptoms, as well as ASD type III, such as thyroglossal sinus to refractive errors of the eye and mitral stenosis. The proband (III.1), a male was between the age range of 12–16, affected with ASD and Thyroglossal sinus. Both parents were assessed for the relevant clinical features but we could not detect any relevant symptoms. Physical examination demonstrated ASD in the patients (Table 1). The family history examination clarified that the patient II2 has the same condition. The II.4 sample, in spite of carrying the mutation, indicated no obvious phenotype implying the reduced penetrance in this condition. All family members were recruited for further physical examination and all gathered records have been reported in Table 1.

### Genetic analysis

**Fig. 1:**
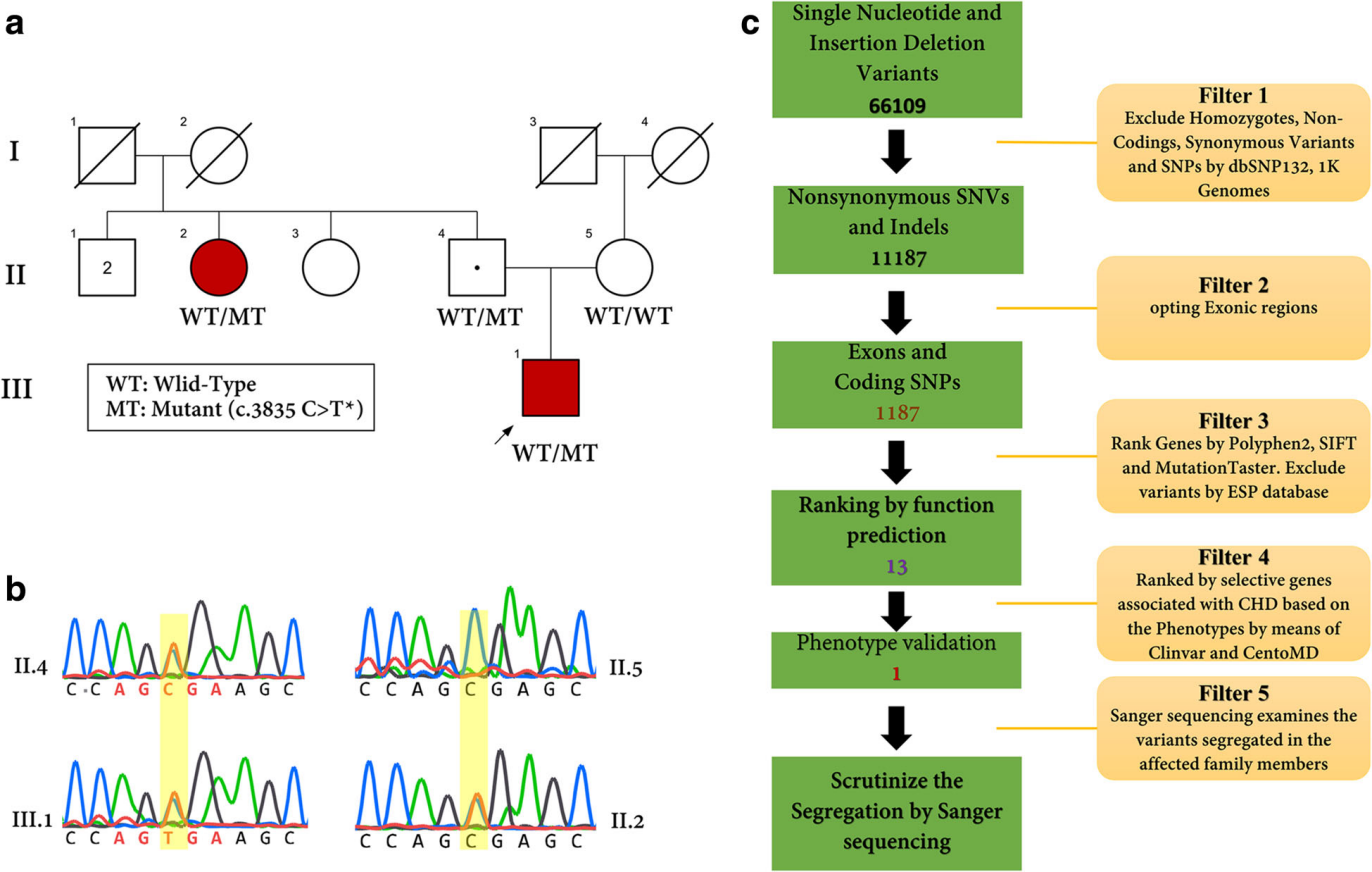
Pedigree, chromatograms and filtering procedures in the SH1190831 family. **a** Pedigree of SH1190831 family is comprised of three generations. The squares and circles indicate males and females, respectively. The arrow appoints the proband of the family. The mutation, c.3835C > T in *MYH6,* has been demonstrated that segregated in this family. **b** Sequence chromatogram showing heterozygote state of the nucleotide sequence of *MYH6 in* c.3835C > T*.* **c** Schematic representation of filtering strategies that applied in this research. The filtering process was applied according several strategies which are demonstrated in the schematic representation. For more investigation, we reevaluated the filtering steps by regard to this fact that the disease could be engendered by autosomal recessive; however, we could not detect any relevant variants according to this supposition

It is postulated that the pedigree may represent an autosomal dominant inheritance with reduced penetrance. To elucidate the underlying genetic cause (s), genomic DNA was obtained from the patient and analyzed by whole exome sequencing (WES). R1279X mutation was confirmed by Sanger sequencing (Fig. 1b).

The detected SNVs and deletion/insertions were analyzed by several filtering methods. 66,109 variants were found in the exome of the proband after alignment and SNV calling. After several exclusion processes by using of dbSNP132, 1000 Genomes Project, Exome Sequencing Project (ESP), and ExAc databases, thirteen variants were identified and then prioritized by patients’ phenotype. Eventually, with the patient’s phenotypes, only one relevant variant was identified that shared by two affected and one carrier family members (II2, III1, II4) but not observed in other healthy parent or normal control (II5).

In the same statement, of the 1187 variants, 13 were ranked using three database tools (Provean, Mutation Taster, Sift) and finally, among the thirteen variants, a unique variant was opted as a pathogenic mutation of this unique family based on patient’s phenotype by utilizing CentoMD (https://www.centogene.com) and ClinVar. (https://www.ncbi.nlm.nih.gov/clinvar/) (Fig. 1c).

Samples from the available members of the SH1190831 family were subjected to Sanger sequencing to confirm the candidate variant of *MYH6* gene.

To find the main cause of CHD in the proband by known genetic mutation (s), based on proband phenotype, we especially focused on the 42 genes that have critical roles in CHD etiology and revised our strategies with a filter of pertinent variants in these genes (Additional file 1: Table S1). The single patient analysis concentrated on the possibility of a known causative gene that underlies CHD.

## Discussion

Atrial septal defect (ASD), a persistent interatrial communication, is one of the common congenital abnormalities occurring in various forms consists of ostium secundum (ASD type II, ~ 75% of cases), ostium primum (ASD type I, 15–20%), sinus venosus (ASD type III, 5–10%), and rarely, coronary sinus defects [15]. ASDs, based on the defected gene, have been classified into several groups. The mutations in various genes have been associated with atrial septal defects, for instance, mutations in *NKX2*–5, *GATA4*, *TBX5*, and *MYH6* [16].

**Fig. 2:**
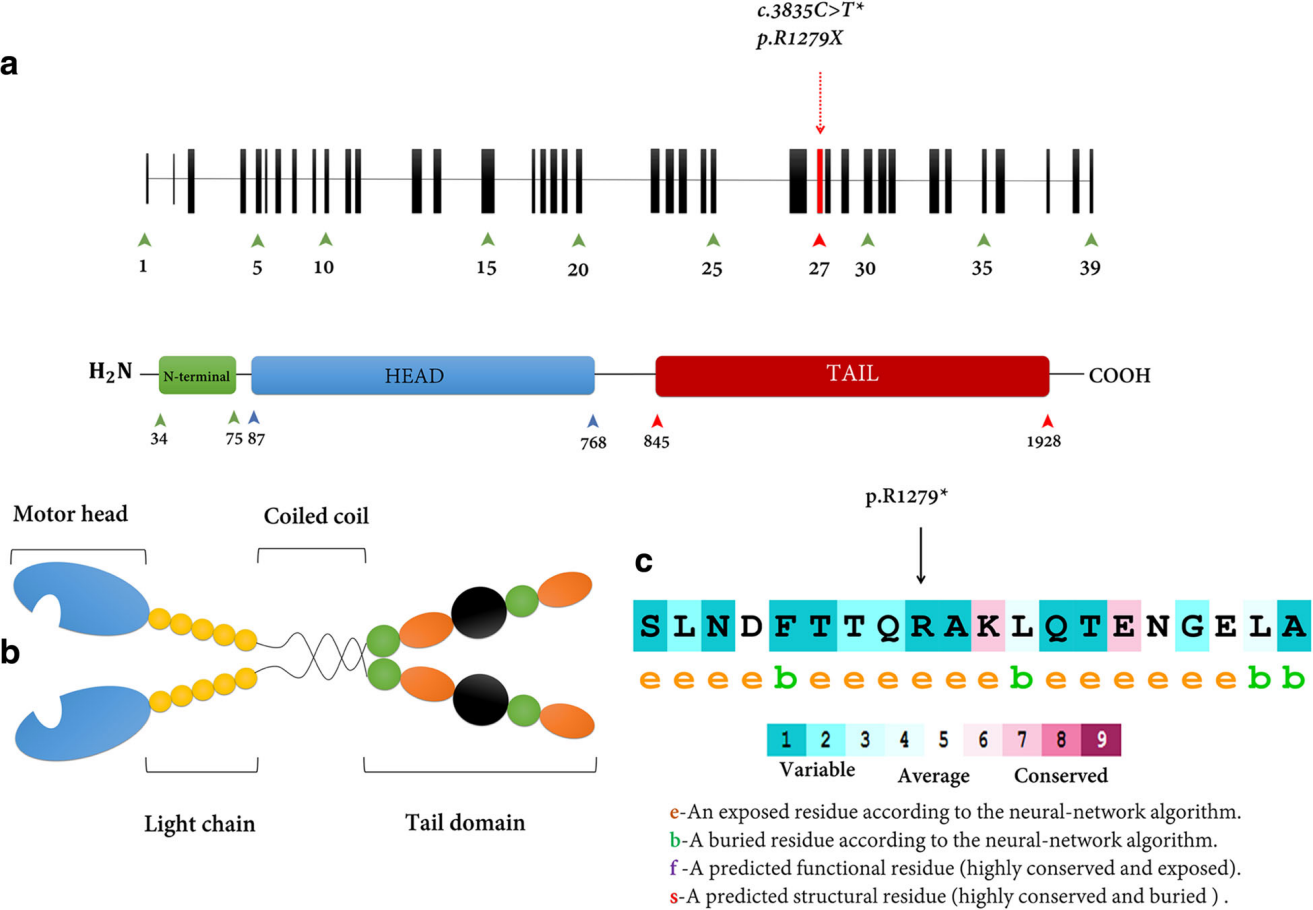
Schematic structure of *MYH6* and Myosin VI protein domains. **a** The *MYH6* gene is located on chromosome 14q12 and composed of 39 exons. *MYH6* codes a protein that comprised of 1928 amino acids. The myosin heavy chain-α (MHC- α) is a hexameric protein (shown by red arrows). The upper red arrow exhibits the position of the mutation, c.3835C > T found in this study. This mutation is located in exon 27 or tail domain region of myosin VI protein. **b** Myosin VI is composed of four various parts. The head domain (blue), Motor Head, binds to actin filaments and hydrolyze ATP; Dimerization is mediated by α-helical coiled-coil domain (Yellow); Tail domain plays a critical role in binding to target proteins. The identified mutation, R1279X, predicted influence on Myosin VI ability to bind a cargo, and it is responsible to emerge a wide phenotype range in these patients. **c** The amino acid sequence MYH6 (p.R1279) colored based on conservation scores by ConSurf database

It has been identified that there are at least 35 classes of molecular motors into the myosin superfamily that move along actin filaments [17]. Several studies have described various functions for Myosin VI such as membrane trafficking, endocytosis, organizing and stabilizing the actin cytoskeleton and playing a material role in inner-ear hair cells [18–20]. Myosin VI is the merely class of myosin that known to move toward the minus-end of actin filaments. Intuitively, dimerization of the myosin can expand its movement along actin filament but it must be noticed that the Myosin VI does not contain a well-defined coiled-coil dimerization domain, suggesting that myosin-VI does not form a constitutive dimer on its own. The *MYH6* gene encodes Myosin heavy chain, α isoform (MHC-α) in human Myosin VI [21]. This protein has several important domains such as head domain, IQ domain, cargo-binding domain, tail domain and etc. (Fig. 2). The tail domain involves two distinct section: Coiled-coil domain and globular domain. It has been identified that the tail domain has a staple role in interacting with the target, especially uncoated vesicles [22].

NGS and particularly whole exome sequencing techniques have been developed into a robust and cost-effective tool to identify the new variants or genes for rare Mendelian unknown disorders [23–25]. This technique has been used in genetic diagnostics helping to increase the clinical and mutational spectrum of known and unknown diseases [26, 27]. But sometimes it is so difficult to distinguish between pathogenic and benign mutations [28, 29]. Several filtering strategies have been developed to exclude variants that are implausible to cause disease.

In this study, we utilized the WES technique to identify a nonsense mutation at nucleotide 3835 of *MYH6* gene. This mutation is located at the extremely conserved region in *MYH6* gene in Primates, Myosin heavy chain-α isoform (MHC-α), and it is presumed to result in a truncated protein that is associated with Cardiomyopathy and ASD type 3 (OMIM: 614089, 613,251). Previously, it has been reported that the mutations in *MYH6* are associated with late-onset hypertrophic cardiomyopathy, atrial septal defects and sick sinus syndrome [14, 30]. There are numerous reports on the association of *MYH6* mutations and CHD [31].

In the present study, we identified a nonsense variant, c.3835C > T, R1279X, by whole exome sequencing in the coiled-coil region or tail domain of *MYH6* gene. This region mediates interaction with cargo molecules or other myosin subunits. After several staple filtering and annotation processes, to predict whether the variant was deleterious or not, we utilized several databases such as SIFT, Mutation Taster, and Provean. We also analyzed intronic, synonymous, nonsense, missense and frameshift indel changes to predict whether those changes could affect splicing process by influencing on donor or acceptor splice sites, with mutation taster and Neutral Network Splice (NNSplice version 0.9).

It has been illustrated that the reduced penetrance could take the Centre stage in increasing prevalence atrial septal defects in familial form. According to the Sanger sequencing results, the mother of the patient, II.4, is a carrier for R1279X mutation. It could be justified by reduced penetrance. This phenomenon can make genetic counseling more challenging because of the difficult interpretation of a person’s family medical history and prediction the risk of passing a genetic condition to offspring.

R1279X mutation could increase the truncated proteins in the cell and, as a result, cell should prevent this process by Nonsense-mediated decay response (NMD response) which is increasingly appreciated as one of the central mechanisms of RNA surveillance, with a big deal role not only in physiological control of gene expression but also in modulating defects and acquired genetic diseases. NMD could confront the cells with reduced amounts of the protein which has known haploinsufficiency (i.e., reduced amounts of protein due to a mutant or null allele) [32]. In nutshell, NMD must be considered when the functional effect of the premature termination codon (PTC). It has been identified that the haploinsufficiency has a staple role in the pathogenesis of cardiovascular diseases [33, 34]. Based on the nonsense mutation influence on proteins, we propose haploinsufficiency as a predicted mechanism of pathogenesis of c.3835C > T, R1279X mutation in the patients, but more studies need to uncover the exact mechanism of CHD pathogenicity.

Our result indicates that this nonsense mutation (R1279X) in *MYH6* might be the genetic cause of congenital heart disease. Our study confirms that the *MYH6* gene has an important role in heart functions but we recommend the applying animal modeling to scrutinize the distinctive role of this mutation.

## Conclusions

For the first time, we identified a nonsense mutation, c.3835C > T, R1279X, in *MYH6* gene as a possible cause of CHD in an Iranian family. This finding will increase our knowledge about the aetiology of this rare condition by effective clarification of the causative gene mutations and will enhance the mutational spectrum of CHD and should consider in the diagnosis of these diseases.

## Additional file

Additional file 1: **Table S1.** A comprehensive menu for CHD curated for clinical scenarios. (DOCX 12 kb)

## Abbreviations

ASD: Atrial septal defect
CCS: Consensus Coding Sequences
CHD: Congenital Heart Disease
DCM: dilated cardiomyopathies
ECG: 12-lead Echocardiogram
ESP: Exome Sequencing Project
HCM: hypertrophic cardiomyopathies
HTS: high throughput sequencing
UCG: Ultrasonic cardiogram
WES: Whole-Exome sequencing
