# Supplementary material for: Whole-exome sequencing identifies R1279X of MYH6 gene to be associated with congenital heart disease
Source: BMC Cardiovasc Disord. 2018 Jul 3;18:137. doi: 10.1186/s12872-018-0867-4 (PMC6029398; doi:10.1186/s12872-018-0867-4)
Supplement: Supplementary file 1 — Table S1. A comprehensive menu for CHD curated for clinical scenarios. (DOCX 12 kb) [file 12872_2018_867_MOESM1_ESM.docx]

| Table S1. **A comprehensive menu for CHD curated for clinical scenarios.** | |
| --- | --- |
| **Condition** | **Genes** |
| **Congenital Heart Disease** | *ACTC1, ACVR2B, ALMS1, BCOR, BRAF, CBL, CHD7, CRELD1, ELN, FOXH1, GATA4, GATA6, GDF1, GJA1, GPC3, HAND1, HRAS, JAG1, KRAS, LEFTY2, MAP2K1, MAP2K2, MED13L, MEIS2, MYH6, NKX2-5, NKX2-6, NODAL, NOTCH1,NR2F2, NRAS, NSD1, PTPN11, RAF1, RIT1, SHOC2, SMAD6, SOS1, TBX1, TBX5, ZFPM2, ZIC3* |
